# Plasma fractionation in Latin America: a path towards sovereignty and sustainability in the supply of plasma-derived medicines

**DOI:** 10.17843/rpmesp.2026.433.15418

**Published:** 2026-08-31

**Authors:** Andrea C. Zucchi, Silvina del V. Druetta

**Affiliations:** 1 Laboratorio de Hemoderivados, Universidad Nacional de Córdoba, Córdoba, Argentina. Universidad Nacional de Córdoba Laboratorio de Hemoderivados Universidad Nacional de Córdoba Córdoba Argentina

**Keywords:** Plasma, Latin America, Plasma-Derived Medicinal Products, Access to Essential Medicines and health technologies

## Abstract

The supply of plasma-derived medicinal products (PDMPs) is a critical global challenge, especially in low- and middle-income countries. In Latin America, thousands of liters of plasma are discarded annually. This work proposes alternative and concrete strategies to initiate plasma fractionation in Latin America. The Hemoderivatives Laboratory of the National University of Córdoba (LHUNC) has implemented a successful regional cooperation model with Uruguay, Chile, and Paraguay. This exchange system allows countries to deliver plasma and receive PDMPs without the need to transfer foreign currency, overcoming barriers such as the lack of national blood plans or low initial collection volumes. In conclusion, the LHUNC experience demonstrates that plasma utilization through proximity alliances is an ethical and financially viable alternative to achieve health sovereignty and reduce regional vulnerability to global market volatility.

## INTRODUCTION

The availability of plasma-derived medicinal products (PDMPs) is a critical global challenge, especially in low- and middle-income countries, where scarcity is widespread ^1^. PDMPs are necessary medicines for the treatment of congenital, rare, or acquired diseases, with intravenous immunoglobulin (IVIG) currently being the most demanded product. The World Health Organization (WHO) has classified several of them as essential medicines ^2^, with few or no alternative treatments.

The demand for these medicines, particularly IVIG, grows at rates higher than the growth rate of plasma, the starting material for these medicines ^3^. Furthermore, there is a high dependence on a few producing countries, with the United States being the main supplier worldwide ^4^^,^^5^. This generates problems of scarcity and access to its derived products, with the consequent risk of shortage. This problem has led to plasma being defined as a strategic resource of national States, comparable to water ^6^. An example of the programs being established in high-income countries is the so-called «Supply» ^7^, a project promoted by the European Union (EU) whose objective is to increase and strengthen the resilience of plasma collection in the EU to enable a stable and adequate supply of PMDPs.

Paradoxically, millions of liters of plasma are discarded annually in middle and low-income countries ^8^, which is also evident in Latin America, where only six countries fractionate their plasma obtaining PMDPs: Argentina, Brazil, Chile, Cuba, Paraguay, and Uruguay. In this context, the present work aims to propose alternatives for Latin American countries to take advantage of the plasma they currently discard, taking the LHUNC's experience as a reference.

### Methodological approach

The work was based on an analysis of the LHUNC experience in the countries with which it has agreements, identifying the barriers that hindered plasma fractionation and the alternatives implemented to overcome them.

The performance indicators of the agreements for each country were obtained from the LHUNC records. The analysis period comprised from the start of plasma contribution, according to the date of each agreement, until December 2024.

### Regional challenge: barriers to plasma fractionation in Latin America

The WHO issued guidance in 2021 ^1^ to promote the fractionation of plasma obtained in each country, with the aim of ensuring a supply of PMDPs that is safe, stable, sufficient, and ethically obtained.

However, the implementation of these recommendations faces multiple barriers. Among the main challenges in the region are those described by WHO, such as the lack of a coordinated national blood system, non-compliance with Good Manufacturing Practices, inadequate infrastructure for transport and storage, the absence or weakness of regulatory oversight, and the fragmentation of blood collection systems, among others ^9^. These limitations often prevent donated plasma from being suitable for fractionation, which contributes to maintaining dependence on imports and limits the availability of vital medicines.

### The guidance of WHO and the proposed process

The WHO proposes a logical process to implement plasma fractionation ^1^, which seeks to transform discarded plasma into PMDP (table 1).

**Table 1:** Steps in capacity building and decision-making for a plasma fractionation program from domestically produced plasma.

	Stage	Description
1	Evaluation of local clinical needs.	Diagnosis of patients. Determination of quantity and types of necessary products. Identification of priority plasma products. Unification of clinical guidelines.
2	State of the blood and plasma collection system.	Existence of a national blood program. Procedures for obtaining blood and plasma. Compliance with good manufacturing practices.
3	Status of the national regulatory system.	Requirements for quality and safety. Inspection Procedures of the Centers. Procedures for plasma authorization.
4	Evaluation of the possibility of plasma fractionation.	Analysis of economic aspects. Selection of technological approaches and products to develop. Necessary regulatory measures.

Source: Own elaboration based on: Guidance for increasing the supply of plasma-derived medicinal products in low- and middle-income countries through fractionation of domestically obtained plasma. WHO/PAHO. 2021.

The implementation of this process requires a strong commitment from public health authorities, which is not always fully present due to the need to address other health emergencies, such as dengue, avian influenza, or chikungunya outbreaks, due to economic restrictions, or due to structural deficiencies related to technical and management capacity ^1^^,^^3^^,^^10^.

### Alternative strategies for plasma utilization for the production of PMDPs in Latin America

Despite these limitations, there are concrete actions that can be carried out in Latin America to leverage plasma for fractionation, even if not all ideal conditions are met at the beginning.

*Evaluation of local clinical needs*

An initial step may be to know the needs for the most demanded blood-derived medicines, mainly human serum albumin (HSA) and intravenous/subcutaneous immunoglobulin G (IVIG/SCIG). A preliminary analysis of the needs for these medicines may constitute a start for the plasma fractionation process.

*Status of the System for Collection of Blood and Plasma*

It is essential to ensure the implementation in blood establishments of Good Manufacturing Practices (GMP), Good Laboratory Practices (GLP), and that they have standardized procedures for blood and plasma collection ^1^. However, this requirement is not always met in all blood establishments of a country. Therefore, it is recommended to have a national blood plan (NBP) that ensures compliance with these requirements.

However, there are blood establishments that meet such requirements. Their survey may arise within the framework of an NBP, if one exists, or through the participation of non-profit organizations such as national universities, scientific associations, or patient associations. This body should be the one to present the identified needs and possible alternatives to the national health authorities. In this process, it is important to have the collaboration of the potential fractionator.

*Status of the national regulatory system*

Regulatory authorities have not always defined the requirements for plasma for fractionation, nor intervened in the authorization of blood establishments as suppliers. On the other hand, every pharmaceutical industry of blood products has defined the requirements for its own operation, and those related to plasma used as raw material ^11^. In Argentina, the National Administration of Medicines, Food and Medical Technology (ANMAT) established in 2012 the requirements for a blood establishment to be a plasma supplier for the industry ^12^^,^^13^.

The transmission of these requirements to other countries can be the driving force for national or regional regulations ^14^.

*Possibility to perform plasma fractionation: economic aspects and contracting models*

When local fractionation is not possible, different modalities exist, such as fractionation contracts ^1^^,^^15^ and plasma for plasma-derived product exchange agreements.

In the region, there are three non-profit plasma derivatives plants. Two of them, located in Argentina and Cuba, are operational. The third one, in Brazil, is under construction and is expected to start production in 2027. Meanwhile, Brazil maintains a fractionation contract with a multinational company ^16^.

These plants, by being located in the region, close to the plasma source sites ^17^, facilitate logistics. Furthermore, they do not require the provision of large volumes of plasma (10,000-50,000 liters annually) demanded by large multinationals ^1^^,^^18^^,^^19^. In this way, countries can progressively develop their capabilities for plasma production and storage for fractionation, while supplying plasma and receiving plasma-derived medicines. The differences between the fractionation contract, such as the one maintained by Brazil, and the plasma-for-hemoderivatives exchange agreement model proposed by LHUNC are shown in table 2.

**Table 2:** Contract models for plasma fractionation by third parties.

Contract Model	Description
1. Fractionation Contract	Country contributes plasma to a foreign fractionation plant.
	The fractionation plant:
	manufactures the medicines stipulated in the contract in the stipulated quantities.
	delivers 100% of the production to the plasma-supplying country.
	charges in foreign currency for the medicine production service
2. Exchange Agreement	Country contributes plasma to a foreign fractionation plant.
	The fractionation plant:
	produces the drugs established in the agreement.
	delivers a quantity (defined in the agreement) of the production obtained to the plasma-supplying country.
	retains part of the production as cost recovery for transport logistics of plasma and drugs, and for their production (no foreign exchange outflow).

Source: Own elaboration.

An economic evaluation that estimates the savings generated by medicines obtained through these models can justify their implementation before health authorities, promote their commitment, and encourage work towards a complete and systematic process.

### LHUNC experience: a regional model

In this context, the experience carried out by LHUNC is significant for the region. The laboratory was created in 1965 as a unit of the National University of Córdoba (UNC) and operates as a non-profit pharmaceutical industry, dedicated to the production of plasma-derived medicines.

Given the absence of an NBP in Argentina during those years, the initiation of fractionation required LHUNC to assume a national leadership role. Thus began the development and support of the country's blood banks, communicating quality requirements and training in GMP and quality management systems.

On the other hand, given the surplus fractionation capacity, and the geographic proximity to other countries in the region, the LHUNC established exchange agreements with Uruguay (1985), Chile (1994), and Paraguay (2014).

Currently, Uruguay does not have a NBP, which can be verified on the website of the Ministry of Health (https://www.gub.uy/ministerio-salud-publica/). The execution of the agreement with the LHUNC was commissioned by the Ministry of Public Health to the National Blood Service (SNS), which created the Plasma Pre-processing Program to manage the activities necessary for its implementation.

In Chile, the agreement began in 1994 with the National University of Chile, since at that time the Ministry of Health could not assume its management. In 2012, the ownership of the agreement was transferred from the National University of Chile to the health services of the country's health regions. Chile still does not have a NBP. Since 2008, there has been a National Transfusion Medicine Coordination (CNMT), which coordinates and supervises the execution of the agreement.

In Paraguay, the only agreement managed from the outset with the NBP was established. During the first year, only one public production center was a plasma supplier. The joint work between the NBP and the LHUNC has managed to incorporate new public and private blood establishments into the agreement. The main indicators of the performance of the agreements with the three countries, from the reception of a first plasma shipment until December 2024, are shown in Table 3.

**Table 3:** Evolution of international exchange agreements.

Country	Performance descriptors of the agreement
Uruguay	% increase in absolute liters of plasma contributed since the first year of plasma shipment (1989) to December 2024: 1912%.
	% increase in HSA received by agreement (period 1989-2024): 4876%
	% increase in IVIG received by agreement (period 1998-2024): 832% (Note: at the beginning of the agreement with Uruguay, IVIG was not yet produced; it was included in 1998).
	Current medications (2026) under agreement: HSA, IVIG, Antihemophilic Factor VIII (FVIII), Prothrombin Complex (PC), and Antithrombin III (ATIII).
Chile	% increase in absolute liters of plasma contributed from the first year of plasma shipment (1994) to December 2024: 2064%
	% increase in HSA received by agreement (period 1989-2024): 4476%
	% increase IVIG received by agreement (period 1994-2024): 6279%
	Current medications (2026) under agreement: HSA, IVIG, Antihemophilic Factor VIII (FVIII).
Paraguay	% increase in absolute liters of plasma contributed from the first year of plasma shipment (2015) to December 2024: 541%
	% increase of HSA received under agreement (period 2015-2024): 531%
	Increase IVIG received by agreement (period 2015-2024): 477%
	Current medications (2026) under agreement: HSA, IVIG, Antihemophilic Factor VIII (FVIII), Prothrombin Complex (PC), and Antithrombin III (ATIII).

Source: Own elaboration from LHUNC data.

As observed in table 3, all countries with current agreements have increased both plasma contribution and the reception of medicines manufactured from this raw material. However, the increase in the quantity of medicines received is not directly proportional to the increase in the volume of plasma contributed, due to various factors that have varied throughout the analyzed period. Among these factors, a larger volume of plasma per shipment reduces logistics and storage costs, and improves the efficiency of the production process, which allows offering more favorable conditions to participating countries. Likewise, the terms of the agreements adapt to the evolution of each country's PMDPs needs. For example, during the initial stages, most countries show a greater demand for HSA than for IVIG.

Figure 1 shows the evolution of plasma provision to the industry by the three countries (Uruguay, Chile, and Paraguay), as well as the main milestones that explain its development. It should be noted that, the initial annual plasma contribution of the three countries was less than 2000 L/year. The observed growth reflects the progressive development of capacities in each of the countries.

**Figure 1:**
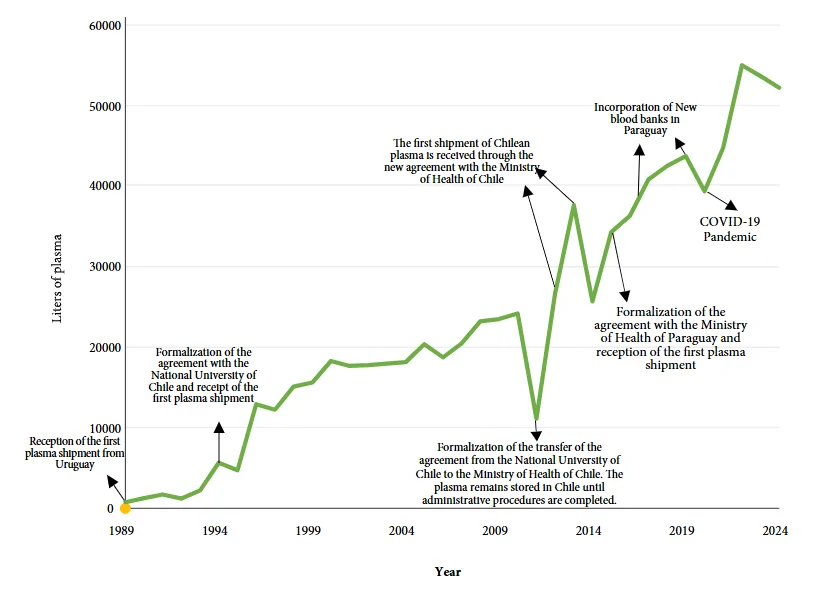
Evolution of the total plasma contribution for fractionation from Uruguay, Chile and Paraguay (period: 1989-2024.

Source: own elaboration based on data from the Blood Derivatives Laboratory of the National University of Córdoba.

### Resilience in crises: experience in the COVID-19 pandemic

During the emergency due to the COVID-19 pandemic, the LHUNC identified key risks that compromised plasma reception, as well as the availability of the corresponding PMDPs. Specific strategies were implemented to maximize plasma collection, adapting logistics in the face of the initial border closure and new requirements for national and international transit.

The impact of the pandemic varied among countries. Argentina and Chile, with a high incidence of COVID-19, showed the greatest reductions in plasma shipment (15% and 17%, respectively); Uruguay maintained a high level of provision (96%), and Paraguay even exceeded the 2019 volume, thanks to the incorporation of a new supplier center ^20^. Despite the difficulties, commitments and the provision of PMDPs to the countries under agreement were fully met.

Likewise, various extraordinary events registered in the region, such as emergencies in storage centers or adverse climatic conditions ^21^^,^^22^, have tested the response capacity of the cooperation network. In these contexts, the geographical proximity, direct communication, coordinated efforts, and institutional commitment of the parties allowed for agile reprogramming of logistical activities and acting in solidarity to sustain provision in unforeseen situations.

### Keys to success: explanatory factors that made regional cooperation possible

The explanatory factors of the success of the LHUNC agreement model include:

*Generation of the strategic vision regarding the importance of PMDPs in the country*

This element is indispensable to initiate the procedures that allow the utilization of plasma for fractionation. Identifying a person or institution that generates this vision and the corresponding commitment, allows to overcome the political difficulties that impede progress in an agreement.

*Public and non-profit nature of the LHUNC*

The UNC acts as a strategic articulator that prioritizes public health and health sovereignty over corporate profit. This generates confidence in the system. Furthermore, its university nature has education at its very genesis, which is decisive for supporting blood banks in improvement processes. This, along with the designation of responsible institutions in countries to lead the work in each of them, allowed to cover the absence of a NBP. Likewise, the regulations applicable to Argentine blood establishments allowed the implementation of specific requirements in the countries under agreement, even if national regulatory authorities had not developed their own.

*Flexibility and scale*

Unlike multinationals that require between 10,000 and 50,000 liters annually to operate, this model allowed for initial contributions of less than 2,000 L/year. This facilitated countries developing their capacities progressively.

*Geographic proximity*

Proximity reduces logistic costs and allows direct communication, which is relevant in unforeseen situations. In addition, LHUNC has a fleet of refrigerated trucks for plasma collection in countries with agreements. This proves to be a great strength in the face of unforeseen events that may require changes in scheduled logistics.

*Characteristic specific to the exchange agreement model*

The model proposed by LHUNC does not require currency exchange for the payment of logistics and production services.

*Resilience in the face of crisis*

The model demonstrated robustness during the COVID-19 pandemic, the closure of border crossings, and fires in storage centers, where solidarity cooperation allowed to reschedule activities and sustain the supply.

### Discussion and lessons learned

Access to PMDPs is a challenge for health sovereignty in Latin America, given that plasma is a strategic national resource. Thousands of liters are discarded annually in the region while a critical dependence on imports controlled by a few producing countries persists. The LHUNC experience demonstrates that this paradox can be resolved through a model of regional cooperation and institutional commitment.

While the fractionation contract used by multinationals, and model adopted by Brazil until it had its own operational plant, offers the advantage that 100% of the production returns to the plasma-supplying country, on the other hand, it requires payment in foreign currency and minimum volumes of up to 50,000 liters annually ^16^. In this regard, the LHUNC exchange agreement offers structural advantages for emerging economies, as it allows initial contributions of less than 2,000 liters, and recovers costs by retaining part of the production. This approach not only protects national monetary reserves by avoiding foreign exchange outflow, but also facilitates that countries with fragmented collection systems develop their capacities progressively.

Among the lessons learned, the experience carried out has shown that the absence of a NBP should not paralyze the use of plasma. In cases like Uruguay and Chile, institutional or university leaderships filled structural gaps, using the LHUNC as a driver to implement quality standards and technical training in blood banks, additionally substituting regulatory gaps or absences. In this sense, the identification of actors or institutions that generate a strategic vision in each nation is critical.

Likewise, geographical proximity proved to be a critical resilience factor. This was demonstrated during the COVID-19 pandemic and various climatic or disaster emergencies. Physical proximity and direct communication allowed to sustain the supply of essential medicines in the face of these various situations.

### Study limitations

The present study has some identifiable limitations. One of them refers to the fact that its results derive exclusively from internal records of LHUNC. Furthermore, the experience carried out and the results derived from it cannot be generalized to the other countries in the region, without considering the political and health contexts. On the other hand, the work has focused its scope on the highest demand medications (HSA and IVIG), not addressing the particularities of, for example, coagulation factors (antihemophilic FVIII, CP, ATIII). Finally, although economic savings are mentioned as justification, the study does not delve into cost analysis or market price comparisons in the different countries. Furthermore, it also lacks an analysis of the long-term financial sustainability of the model.

### Final recommendations and conclusions

Firstly, the imperative need to raise awareness among regional stakeholders about the strategic relevance of plasma utilization for PDMPs is highlighted. This action is critical to mitigate the vulnerability of healthcare systems to potential risks of shortages. In this regard, the development of a comprehensive political vision constitutes the indispensable catalyst to overcome existing administrative and technical obstacles. Under this premise, it is recommended that States initiate diagnostic evaluation processes regarding their local clinical needs, with special emphasis on high-demand PDMPs, HSA and IVIG, in order to support technical and political investment before the competent authorities. It should be noted that any intervention aimed at optimizing hemocomponent collection processes and strengthening their biological safety, under GMP standards, represents an essential preparatory phase for the consolidation of a regional PDMP industry.

In the Latin American scenario, the LHUNC model stands as a transformative paradigm that values a previously discarded resource, converting it into essential therapeutic medicines. Its effectiveness lies in the implementation of a flexible and solidarity scheme that strengthens regional autonomy in the face of global market volatility, evidencing that health sovereignty is achievable through proximity alliances. In summary, the plasma for blood products exchange model is consolidated as a viable ethical and financial alternative for middle-income economies that aim to optimize public spending and preserve their foreign currency reserves.
