# Supplementary material for: Plasma fractionation in Latin America: a path towards sovereignty and sustainability in the supply of plasma-derived medicines
Source: Rev Peru Med Exp Salud Publica. 2026 Aug 31;43(3):390–6. doi: 10.17843/rpmesp.2026.433.15418 (PMC13718563; doi:10.17843/rpmesp.2026.433.15418)
Supplement: Translation

# Translation: Fraccionamiento de plasma en Latinoamérica: un camino hacia la soberanía y sostenibilidad en el suministro de medicamentos hemoderivados

**Authors:** Andrea C. Zucchi, Silvina del V. Druetta

**Affiliations:** 1 Laboratorio de Hemoderivados, Universidad Nacional de Córdoba, Córdoba, Argentina.

**Keywords:** Plasma, Latinoamérica, Productos Medicinales Derivados del Plasma, Acceso a Medicamentos Esenciales y Tecnologías Sanitarias

## Abstract

El suministro de productos medicinales derivados del plasma (PMDP) es un desafío crítico a nivel global, especialmente en países de ingresos medianos y bajos. En América Latina, miles de litros de plasma se descartan anualmente. Este trabajo propone estrategias alternativas y concretas para iniciar el fraccionamiento de plasma en Latinoamérica. El Laboratorio de Hemoderivados de la Universidad Nacional de Córdoba (LHUNC) ha implementado un exitoso modelo de cooperación regional con Uruguay, Chile y Paraguay. Este sistema de intercambio permite a los países entregar plasma y recibir PDMP sin necesidad de girar divisas, superando barreras como la falta de planes nacionales de sangre o bajos volúmenes iniciales de recolección. Como conclusión, la experiencia del LHUNC demuestra que el aprovechamiento del plasma mediante alianzas de proximidad es una alternativa ética y financieramente viable para alcanzar la soberanía sanitaria y reducir la vulnerabilidad regional ante la volatilidad del mercado global.

## INTRODUCCIÓN

La disponibilidad de productos medicinales derivados del plasma (PMDP) es un desafío crítico a nivel global, especialmente en países de ingresos medianos y bajos, donde la insuficiencia es generalizada ^1^. Los PMDP son medicamentos necesarios para el tratamiento de enfermedades congénitas, raras o adquiridas, siendo en la actualidad la inmunoglobulina endovenosa (IGEV) el producto de mayor demanda. La Organización Mundial de la Salud (OMS) ha catalogado a varios de ellos como medicamentos esenciales ^2^, con pocos o ningún tratamiento alternativo.

La demanda de estos medicamentos, particularmente la IGEV, crece a tasas superiores a la tasa de crecimiento del plasma, material de partida de estos medicamentos ^3^. Además, existe una alta dependencia de pocos países productores, siendo Estados Unidos el principal proveedor a nivel mundial ^4^^,^^5^. Esto genera problemas de escasez y acceso a los productos derivados del mismo, con el consiguiente riesgo de desabastecimiento. Esta problemática ha llevado a que el plasma haya sido definido como un recurso estratégico de los Estados nacionales, equiparable con el agua ^6^. Un ejemplo de los programas que se están estableciendo en los países de altos ingresos es el denominado **«**Supply**»**^7^, proyecto impulsado por la Unión Europea (UE) cuyo objetivo es aumentar y fortalecer la resiliencia de la recolección de plasma en la UE para permitir un suministro estable y adecuado de PMDP.

Paradójicamente, millones de litros de plasma se descartan anualmente en países de medianos y bajos ingresos ^8^, lo cual se evidencia también en América Latina, donde sólo seis países fraccionan su plasma obteniendo PMDP: Argentina, Brasil, Chile, Cuba, Paraguay y Uruguay. En este contexto, el presente trabajo tiene como objetivo proponer alternativas para que los países latinoamericanos aprovechen el plasma que actualmente descartan, tomando como referencia la experiencia del LHUNC.

### Abordaje metodológico

El trabajo se realizó a partir del análisis de la experiencia del LHUNC en los países con los que mantiene convenios, identificando las barreras que dificultaban el fraccionamiento del plasma y las alternativas implementadas para superarlas.

Los indicadores del desempeño de los convenios para cada país se obtuvieron de los registros del LHUNC. El período de análisis comprendió desde el inicio del aporte de plasma, según la fecha de cada convenio, hasta diciembre de 2024.

### Desafío regional: barreras para el fraccionamiento de plasma en Latinoamérica

La OMS emitió una guía en 2021 ^1^ para fomentar el fraccionamiento de plasma obtenido en cada país, con el objetivo de asegurar un suministro de PMDP que sea seguro, estable, suficiente y éticamente obtenido.

Sin embargo, la implementación de estas recomendaciones enfrenta múltiples barreras. Entre los principales desafíos de la región se encuentran los descritos por la OMS, como la falta de un sistema sanguíneo nacional coordinado, el incumplimiento de las Buenas Prácticas de Fabricación, la infraestructura inadecuada para el transporte y almacenamiento, la ausencia o debilidad de la supervisión regulatoria, y la fragmentación de los sistemas de recolección de sangre, entre otros ^9^. Estas limitaciones impiden muchas veces que el plasma donado sea apto para el fraccionamiento, lo que contribuye a mantener la dependencia de las importaciones y limita la disponibilidad de medicamentos vitales.

## La orientación de la OMS y el proceso propuesto

La OMS propone un proceso lógico para implementar el fraccionamiento de plasma ^1^, el cual busca transformar el plasma descartado en PMDP (tabla 1).

**Tabla 1:** Pasos en la creación de capacidad y la toma de decisiones para un programa de fraccionamiento del plasma a partir del plasma producido en el país.

Etapa		Descripción
1.	Evaluación de las necesidades clínicas locales.	Diagnóstico de pacientes. Determinación de cantidad y tipos de productos necesarios. Identificación de productos de plasma prioritarios. Unificación de directrices clínicas.
2.	Estado del sistema de recolección de sangre y plasma.	Existencia de un programa nacional de sangre. Procedimientos de obtención de sangre y plasma. Cumplimiento de buenas prácticas de manufactura.
3.	Estado del sistema regulatorio nacional.	Requisitos de calidad y seguridad. Procedimientos de inspección de los centros. Procedimientos de autorización del plasma.
4.	Evaluación de la posibilidad de fraccionamiento del plasma.	Análisis de aspectos económicos. Selección de enfoques tecnológicos y productos a desarrollar. Medidas regulatorias necesarias.

Fuente: Elaboración propia basada en: Orientación para aumentar el suministro de productos medicinales derivados del plasma en los países de ingresos medianos y bajos mediante fraccionamiento del plasma obtenido en el propio país. OMS/OPS. 2021.

La implementación de este proceso requiere de un fuerte compromiso de las autoridades de salud pública, el cual no siempre está plenamente presente por la necesidad de atender otras urgencias sanitarias, como brotes de dengue, influenza aviar o chikungunya, por restricciones económicas o por deficiencias estructurales relacionadas con la capacidad técnica y de gestión ^1^^,^^3^^,^^10^.

### Estrategias alternativas para el aprovechamiento de plasma para la elaboración de PMDP en Latinoamérica

A pesar de estas limitaciones, existen acciones concretas que pueden llevarse adelante en Latinoamérica para aprovechar el plasma para fraccionamiento, incluso si no se cumplen todas las condiciones ideales al inicio.

*Evaluación de las necesidades clínicas locales*

Un paso inicial puede ser conocer las necesidades de los medicamentos hemoderivados de mayor demanda, principalmente albúmina sérica humana (ASH) y la inmunoglobulina G endovenosa/subcutánea (IGEV/SC). Un análisis preliminar de las necesidades de estos medicamentos puede constituir un inicio para el proceso de fraccionamiento del plasma.

*Estado del sistema de recolección de sangre y plasma*

Es indispensable garantizar la implementación en los establecimientos de sangre de las Buenas Prácticas de Manufactura (BPM), las Buenas Prácticas de Laboratorio (BPL), y que cuenten con procedimientos estandarizados para la obtención de sangre y plasma ^1^. Sin embargo, no siempre se cuenta con este requisito en todos los establecimientos de sangre de un país. Por ello, se recomienda contar con un plan nacional de sangre (PNS) que asegure el cumplimiento de estos requisitos.

No obstante, existen establecimientos de sangre que reúnen tales requerimientos. Su relevamiento puede surgir en el marco de un PNS, si lo hubiera, o mediante la participación de organizaciones sin fines de lucro como universidades nacionales, asociaciones científicas o asociaciones de pacientes. Este organismo debiera ser el que presente las necesidades identificadas y las posibles alternativas a las autoridades sanitarias nacionales. En este proceso es importante contar con la colaboración del potencial fraccionador. 

*Estado del sistema regulatorio nacional*

No siempre las autoridades regulatorias han definido los requerimientos del plasma para fraccionamiento, ni intervenido en la autorización de los establecimientos de sangre como proveedores. Por otra parte, toda industria farmacéutica de hemoderivados tiene definidos los requerimientos para su propio funcionamiento, y aquellos relacionados al plasma utilizado como materia prima ^11^. En Argentina, la Administración Nacional de Medicamentos, Alimentos y Tecnología Médica (ANMAT) estableció en 2012 los requerimientos para que un establecimiento de sangre sea proveedor de plasma para la industria ^12^^,^^13^.

La transmisión de estos requerimientos a otros países, puede ser el motor movilizador de una normativa nacional o regional ^14^.

*Posibilidad de realizar el fraccionamiento del plasma: aspectos económicos y modelos de contratación*

Cuando el fraccionamiento local no es posible, existen diferentes modalidades como los contratos de fraccionamiento ^1^^,^^15^ y los convenios de intercambio de plasma por hemoderivados.

En la región existen tres plantas de derivados plasmáticos sin fines de lucro. Dos de ellas, ubicadas en Argentina y Cuba, se encuentran operativas. La tercera, en Brasil, está en construcción, y prevé iniciar su producción en 2027. Mientras tanto, Brasil mantiene un contrato de fraccionamiento con una empresa multinacional ^16^.

Estas plantas, al encontrarse en la región, cercanas a las fuentes de origen del plasma ^17^, facilitan la logística. Además, no requieren la provisión de grandes volúmenes de plasma (10 000-50 000 litros anuales) demandados por las grandes multinacionales ^1^^,^^18^^,^^19^. De esta manera, los países pueden ir desarrollando progresivamente sus capacidades de producción y almacenamiento de plasma para fraccionamiento, mientras aportan plasma y reciben medicamentos derivados de este. Las diferencias entre el contrato por fraccionamiento, como el que mantiene Brasil, y el modelo de convenio de intercambio plasma por hemoderivados propuesto por el LHUNC se muestran en tabla 2.

**Tabla 2:** Modelos de contrato para el fraccionamiento de plasma por terceros.

Modelo de contrato		Descripción	
1.	Contrato por fraccionamiento	País aporta plasma a una planta fraccionadora extranjera.	
		La planta fraccionadora:	
		a)	Elabora los medicamentos establecidos en el contrato en las cantidades establecidas.
		b)	Entrega el 100% de la producción al país proveedor del plasma.
		c)	Cobra en divisas el servicio de producción de los medicamentos.
2.	Convenio de intercambio	País aporta plasma a una planta fraccionadora extranjera.	
		La planta fraccionadora:	
		a)	Elabora los medicamentos establecidos en el convenio.
		b)	Entrega una cantidad (definida en el convenio) de la producción obtenida, al país proveedor del plasma.
		c)	Retiene parte de la producción como recupero de costos de logística de transporte de plasma y medicamentos, y de producción de los mismos (no hay giro de divisas).

Fuente: elaboración propia.

Una evaluación económica que estime el ahorro generado por los medicamentos obtenidos mediante estos modelos puede justificar su implementación ante las autoridades sanitarias, favorecer su compromiso y fomentar el trabajo hacia un proceso completo y sistemático.

### Experiencia del LHUNC: un modelo regional

En este contexto, la experiencia realizada por el LHUNC resulta significativa para la región. El laboratorio se creó en 1965 como una dependencia de la Universidad Nacional de Córdoba (UNC) y funciona como una industria farmacéutica sin fines de lucro, dedicada a la producción de medicamentos derivados del plasma.

Ante la ausencia de un PNS en Argentina durante esos años, el inicio del fraccionamiento requirió que el LHUNC asumiera un papel de liderazgo a nivel nacional. De esta manera comenzó el desarrollo y acompañamiento de los bancos de sangre del país, comunicando los requerimientos de calidad y capacitando en BPM y sistemas de gestión de calidad.

Por otra parte, dada la capacidad de fraccionamiento excedente, y la proximidad geográfica con otros países de la región, el LHUNC entabló convenios de intercambio con Uruguay (1985), Chile (1994) y Paraguay (2014).

Actualmente, Uruguay no cuenta con un PNS, lo cual puede verificarse en la página web del Ministerio de Salud (https://www.gub.uy/ministerio-salud-publica/). La ejecución del convenio con el LHUNC fue encargada por el Ministerio de Salud Pública al Servicio Nacional de Sangre (SNS), que creó el Programa de Preprocesamiento de Plasma para gestionar las actividades necesarias para su implementación.

En Chile el convenio se inició en 1994 con la Universidad Nacional de Chile, ya que en ese momento el Ministerio de Salud no podía asumir su gestión. En 2012 se realizó el traspaso de la titularidad del convenio desde la Universidad Nacional de Chile a los servicios de salud de las regiones sanitarias del país. Chile aún no cuenta con un PNS. Desde 2008 existe una Coordinación Nacional de Medicina Transfusional (CNMT), la cual coordina y supervisa la ejecución del convenio.

En Paraguay se dio el único convenio gestionado desde un inicio con el PNS. Durante el primer año, sólo un centro productor público fue proveedor de plasma. El trabajo conjunto entre el PNS y el LHUNC ha logrado incorporar al convenio nuevos establecimientos de sangre públicos y privados. Los principales indicadores del desempeño de los convenios con los tres países, desde la recepción de un primer embarque de plasma hasta diciembre de 2024, se muestran en la tabla 3.

**Tabla 3:** Evolución convenios de intercambio internacionales.

País	Indicadores de desempeño del convenio
Uruguay	Porcentaje de incremento de litros de plasma absolutos aportados desde el primer año de envío de plasma (1989) a diciembre de 2024: 1912%.
	Porcentaje de incremento de ASH recibido por convenio (período 1989-2024): 4876%
	Porcentaje de incremento IGEV recibido por convenio (período 1998-2024): 832% (observación: al inicio del convenio con Uruguay aún no se elaboraba IGEV; la misma fue incluida en 1998).
	Medicamentos actuales (2026) en convenio: ASH, IGEV, factor VIII antihemofílico (FVIII), complejo protrombínico (CP) y antitrombina III (ATIII).
Chile	Porcentaje de incremento de litros de plasma absolutos aportados desde el primer año de envío de plasma (1994) a diciembre 2024: 2064%.
	Porcentaje de incremento de ASH recibido por convenio (período 1989-2024): 4476%.
	Porcentaje de incremento IGEV recibido por convenio (período 1994-2024): 6279%.
	Medicamentos actuales (2026) en convenio: ASH, IGEV, Factor VIII antihemofílico (FVIII).
Paraguay	Porcentaje de incremento litros de plasma absolutos aportados desde el primer año de envío de plasma (2015) a diciembre 2024: 541%.
	Porcentaje de incremento de ASH recibido por convenio (período 2015-2024): 531%.
	Porcentaje de incremento IGEV recibido por convenio (período 2015-2024): 477%.
	Medicamentos actuales (2026) en convenio: ASH, IGEV, factor VIII antihemofílico (FVIII), complejo protrombínico (CP) y antitrombina III (ATIII).

IGEV: inmunoglobulina endovenosa, ASH: albúmina sérica humana.

Como se observa en la tabla 3, todos los países con convenios vigentes han incrementado tanto el aporte de plasma como la recepción de medicamentos elaborados a partir de esta materia prima. Sin embargo, el aumento en la cantidad de medicamentos recibidos no es directamente proporcional al incremento del volumen de plasma aportado, debido a diversos factores que han variado a lo largo del período analizado. Entre estos factores, un mayor volumen de plasma por embarque reduce los costos logísticos y de almacenamiento, y mejora la eficiencia del proceso productivo, lo que permite ofrecer condiciones más favorables a los países participantes. Asimismo, los términos de los convenios se adaptan a la evolución de las necesidades de PMDP de cada país. Por ejemplo, durante las etapas iniciales, la mayoría de los países presenta una mayor demanda de ASH que de IGEV.

En la figura 1 se muestra la evolución de la provisión de plasma a la industria por parte de los tres países (Uruguay, Chile y Paraguay), así como los principales hitos que explican su desarrollo. Cabe destacar que, el aporte de plasma anual inicial de los tres países fue inferior a 2000 L/año. El crecimiento observado refleja el desarrollo progresivo de las capacidades en cada uno de los países.

**Figura 1:** Evolución del aporte total de plasma para fraccionamiento de Uruguay, Chile y Paraguay (período: 1989-2024).

Fuente: elaboración propia a partir de datos del Laboratorio de Hemoderivados de la Universidad Nacional de Córdoba.

### Resiliencia ante crisis: experiencia en la pandemia de COVID-19

Durante la emergencia debida a la pandemia de COVID-19, el LHUNC identificó riesgos clave que comprometían la recepción de plasma, así como la disponibilidad de los PMDP correspondientes. Se implementaron estrategias específicas para maximizar la recolección de plasma, adaptando la logística frente al inicial cierre de fronteras y nuevos requerimientos para el tránsito nacional e internacional.

El impacto de la pandemia varió entre los países. Argentina y Chile, con alta incidencia de COVID-19, mostraron las mayores reducciones en el envío de plasma (15% y 17%, respectivamente); Uruguay mantuvo un alto nivel de provisión (96%), y Paraguay incluso superó el volumen de 2019, gracias a la incorporación de un nuevo centro proveedor ^20^. Pese a las dificultades, se cumplió en su totalidad con los compromisos y la provisión de PMDP a los países bajo convenio.

Asimismo, distintos eventos extraordinarios registrados en la región, como emergencias en centros de almacenamiento o condiciones climáticas adversas ^21^^,^^22^, han puesto a prueba la capacidad de respuesta de la red de cooperación. En estos contextos, la proximidad geográfica, la comunicación directa, las gestiones coordinadas y el compromiso institucional de las partes permitieron reprogramar actividades logísticas con agilidad y actuar de manera solidaria para sostener la provisión ante situaciones imprevistas.

### Claves del éxito: factores explicativos que posibilitaron la cooperación regional

factores explicativos del éxito del modelo de convenio del LHUNC incluyen:

*Generación de la visión estratégica respecto de la importancia de los PMDP en el país*

Este elemento es indispensable para iniciar las gestiones que permitan el aprovechamiento de plasma para fraccionamiento. Identificar una persona o institución que genere esta visión y el compromiso correspondiente, permite superar las dificultades políticas que impiden el avance en un convenio.

*Naturaleza pública y sin fines de lucro del LHUNC*

La UNC actúa como un articulador estratégico que prioriza la salud pública y la soberanía sanitaria sobre el lucro corporativo. Esto genera confianza en el sistema. Además, su ser universitario tiene en su propia génesis la educación, lo cual es determinante para el acompañamiento de los bancos de sangre en los procesos de mejora. Esto y la designación de instituciones responsables en los países para liderar el trabajo en cada uno de ellos, permitió suplir la ausencia de un PNS. Asimismo, la normativa aplicable a los establecimientos de sangre argentinos, permitió la implementación de requerimientos específicos en los países bajo convenio, aunque las autoridades regulatorias nacionales no hubieran desarrollado las propias.

*Flexibilidad y escala*

A diferencia de las multinacionales que exigen entre 10 000 y 50 000 litros anuales para operar, este modelo permitió aportes iniciales inferiores a 2000 L/año. Esto facilitó que los países desarrollaran sus capacidades de forma progresiva.

*Proximidad geográfica*

La cercanía reduce costos logísticos y permite una comunicación directa, relevante ante situaciones imprevistas. A ello se suma que el LHUNC dispone de una flota de camiones refrigerados para la recolección de plasma en los países con convenio. Esta resulta una gran fortaleza ante imprevistos que pueden demandar cambios en la logística programada.

*Característica propia del modelo de convenio por intercambio*

El modelo propuesto por el LHUNC no requiere giro de divisas para el pago de los servicios de logística y producción.

*Resiliencia ante la crisis*

El modelo demostró solidez durante la pandemia de COVID-19, el cierre de pasos fronterizos e incendios en centros de almacenamiento, donde la cooperación solidaria permitió reprogramar actividades y sostener el suministro.

### Discusión y lecciones aprendidas

El acceso a PMDP es un desafío para la soberanía sanitaria en América Latina, dado que el plasma es un recurso estratégico nacional. Miles de litros se descartan anualmente en la región mientras persiste una dependencia crítica de importaciones controladas por pocos países productores. La experiencia del LHUNC demuestra que esta paradoja puede resolverse mediante un modelo de cooperación regional y compromiso institucional.

Si bien el contrato por fraccionamiento utilizado por multinacionales, y modelo adoptado por Brasil hasta contar con su propia planta operativa, ofrece la ventaja de que el 100% de la producción retorna al país proveedor del plasma, exige por otra parte, el pago en divisas y volúmenes mínimos de hasta 50 000 litros anuales ^16^. En este sentido, el convenio de intercambio del LHUNC ofrece ventajas estructurales para economías emergentes, ya que permite aportes iniciales inferiores a 2000 litros, y recupera costos reteniendo parte de la producción. Este enfoque no sólo protege las reservas monetarias nacionales al evitar el giro de divisas, sino que facilita que países con sistemas de recolección fragmentados desarrollen sus capacidades de forma progresiva.

Entre las lecciones aprendidas, la experiencia realizada ha demostrado que la ausencia de un PNS no debe paralizar el aprovechamiento del plasma. En casos como Uruguay y Chile, liderazgos institucionales o universitarios suplieron vacíos estructurales, utilizando al LHUNC como motor para implementar estándares de calidad y capacitación técnica en bancos de sangre, sustituyendo además los vacíos o ausencias regulatorias. En este sentido, la identificación de actores o instituciones que generen una visión estratégica en cada nación resulta crítica.

Asimismo, la proximidad geográfica demostró ser un factor de resiliencia crítico. Esto quedó demostrado durante la pandemia de COVID-19 y diversas emergencias climáticas o de siniestros. La cercanía física y la comunicación directa permitieron sostener el suministro de medicamentos esenciales frente a estas diversas situaciones.

### Limitaciones del estudio

El presente estudio tiene algunas limitaciones identificables. Una de ellas está referida a que sus resultados derivan exclusivamente de registros internos del LHUNC. Además, la experiencia realizada y los resultados que de ella se derivan no se pueden generalizar a los demás países de la región, sin considerar los contextos políticos y sanitarios. Por otra parte, el trabajo se ha centrado en su alcance a los medicamentos de mayor demanda (ASH e IGEV), no abordando las particularidades que tienen, por ejemplo, los factores de coagulación (FVIII antihemofílico, CP, ATIII). Finalmente, si bien se menciona el ahorro económico como justificación, el estudio no profundiza en análisis de costos o comparativas de precios de mercado en los distintos países. Por otra parte, carece además de un análisis sobre la sostenibilidad financiera del modelo a largo plazo.

### Recomendaciones finales y conclusiones

En primera instancia, se destaca la imperativa necesidad de sensibilizar a los actores regionales sobre la relevancia estratégica del aprovechamiento del plasma para la PDMP. Esta acción resulta crítica para mitigar la vulnerabilidad de los sistemas sanitarios frente a potenciales riesgos de desabastecimiento. Al respecto, el desarrollo de una visión política integral constituye el catalizador indispensable para trascender los obstáculos administrativos y técnicos vigentes. Bajo esta premisa, se recomienda que los Estados inicien procesos de evaluación diagnóstica sobre sus necesidades clínicas locales, con especial énfasis en los PDMP de mayor demanda, ASH e IGEV, a fin de sustentar la inversión técnica y política ante las autoridades competentes. Recordar que, cualquier intervención orientada a la optimización de los procesos de obtención de hemocomponentes y al fortalecimiento de su seguridad biológica, bajo estándares de BPM, representa una fase preparatoria esencial para la consolidación de una industria regional de PDMP.

En el escenario latinoamericano, el modelo del LHUNC se erige como un paradigma transformador que valoriza un recurso anteriormente descartado, convirtiéndolo en medicamentos terapéuticos esenciales. Su eficacia radica en la implementación de un esquema flexible y solidario que robustece la autonomía regional frente a la volatilidad del mercado global, evidenciando que la soberanía sanitaria es alcanzable mediante alianzas de proximidad. En suma, el modelo de intercambio de plasma por hemoderivados se consolida como una alternativa ética y financiera viable para economías de ingresos medios que aspiran a optimizar el gasto público y preservar sus reservas de divisas.
